# *Lrig1* expression identifies airway basal cells with high proliferative capacity and restricts lung squamous cell carcinoma growth

**DOI:** 10.1183/13993003.00816-2020

**Published:** 2022-03-31

**Authors:** Laura Succony, Sandra Gómez-López, Adam Pennycuick, Ahmed S.N. Alhendi, Derek Davies, Sarah E. Clarke, Kate H.C. Gowers, Nicholas A. Wright, Kim B. Jensen, Sam M. Janes

**Affiliations:** 1 Lungs for Living Research Centre, UCL Respiratory, University College London, London, UK; 2 Flow Cytometry Facility, Francis Crick Institute, London, UK; 3 Centre for Tumour Biology, Barts Cancer Institute, Queen Mary University of London, London, UK; 4 Biotech Research and Innovation Centre, University of Copenhagen; Novo Nordisk Foundation Center for Stem Cell Biology, DanStem, University of Copenhagen, Copenhagen, Denmark; 5 These authors contributed equally to this work

## Abstract

**Background:**

Lung squamous cell carcinoma (LUSC) accounts for a significant proportion of cancer deaths worldwide, and is preceded by the appearance of progressively disorganised pre-invasive lesions in the airway epithelium. Yet the biological mechanisms underlying progression of pre-invasive lesions into invasive LUSC are not fully understood. *LRIG1* (leucine-rich repeats and immunoglobulin-like domains 1) is downregulated in pre-invasive airway lesions and invasive LUSC tumours and this correlates with decreased lung cancer patient survival.

**Methods and results:**

Using an *Lrig1* knock-in reporter mouse and human airway epithelial cells collected at bronchoscopy, we show that during homeostasis LRIG1 is heterogeneously expressed in the airway epithelium. In basal airway epithelial cells, the suspected cell of origin of LUSC, LRIG1 identifies a subpopulation of progenitor cells with higher *in vitro* proliferative and self-renewal potential in both the mouse and human. Using the N-nitroso-tris-chloroethylurea (NTCU)-induced murine model of LUSC, we find that *Lrig1* loss-of-function leads to abnormally high cell proliferation during the earliest stages of pre-invasive disease and to the formation of significantly larger invasive tumours, suggesting accelerated disease progression.

**Conclusion:**

Together, our findings identify LRIG1 as a marker of basal airway progenitor cells with high proliferative potential and as a regulator of pre-invasive lung cancer progression. This work highlights the clinical relevance of LRIG1 and the potential of the NTCU-induced LUSC model for functional assessment of candidate tumour suppressors and oncogenes.

## Abstract 2

**LRIG1 is lost in development of squamous cell lung cancers. This study shows that LRIG1 marks basal airway progenitor cells with high proliferative potential and regulates progression of pre-invasive squamous cell lung cancer.** https://bit.ly/3AbPtY3

## Introduction

Lung cancer is the main cause of cancer-related deaths worldwide with 2.1 million new cases diagnosed each year [1]. The majority of patients present with late-stage incurable disease [2], therefore, devising strategies for early detection and treatment is key to improving lung cancer outcomes.

85% of lung cancer cases are nonsmall cell lung cancer (NSCLC), of which a third are lung squamous cell carcinoma (LUSC) [3, 4]. LUSC arises in the bronchial epithelium, preceded by the development of progressively disordered pre-invasive lesions, ranging from metaplasia to increasing grades of dysplasia and carcinoma *in situ* (CIS) [5, 6]. Pre-invasive squamous airway lesions are associated with tobacco cigarette smoking, the predominant lung cancer risk factor [5, 7, 8]. Molecular studies have identified genetic, epigenetic and transcriptional changes in pre-invasive lesions [9–13]. However, the biological relevance of these changes in lung cancer development is not understood.

Leucine-rich repeats and immunoglobulin-like domains 1 (LRIG1) is a transmembrane protein that acts as a negative regulator of epidermal growth factor receptor (EGFR) signalling [14, 15]. *LRIG1* is located in chromosome 3p14, a region that is frequently affected by copy number alterations in pre-invasive lung lesions and NSCLC [16, 17]. Loss of heterozygosity of *LRIG1* is seen in 75% of human lung cancer cell lines and low levels of *LRIG1* expression have been correlated with decreased overall survival in patients with NSCLC [18–20]. We have shown previously that both transcript and protein levels of LRIG1 are lower in pre-invasive CIS lung lesions compared to donor-matched healthy epithelial tissue [18], suggesting an early role for LRIG1 during lung carcinogenesis. Here, we investigate the expression of LRIG1 in the normal airway epithelium and examine consequences of its loss-of-function during LUSC development.

## Materials and methods

### Mouse husbandry and experimentation

Animal work was approved by the University College London biological services review committee and carried out in compliance with the UK Home Office procedural and ethical guidelines. The C57BL/6 *Lrig1::eGFP-IRES-CreERT^2^* murine line [21] was backcrossed twice to FVB/N. Mice were maintained in a mixed C57BL/6 and FVB/N background in individually ventilated cages, on a 12-h day/night cycle with access to food and water *ad libitum*. Littermates were distributed in the appropriate experimental or control groups.

### Human tissue samples

Ethical approval was obtained through the National Research Ethics Committee (REC reference 06/Q0505/12). Bronchial samples were taken during autofluorescence bronchoscopy from areas of normal bronchial epithelium. Bronchial brushes were used directly for flow cytometry and tissue biopsies frozen in optimal cutting temperature (OCT) compound (Tissue-Tek 4583).

### Flow cytometry

Flow cytometry was performed on a Fortessa cell analyser (BD Biosciences) and cell sorting on a FACSaria (BD Biosciences). For cell-cycle analysis, live cells were incubated with 1 μg·mL^−1^ Hoechst 33342 for 30 min at 37°C prior to antibody staining. Reagents used are indicated in supplementary table S1. Data was analysed using FlowJo 10.0.6 (Tree Star).

### NTCU-induced LUSC model

The dorsal fur of 6-week-old female mice was shaved and 75 μL of 0.013 M N-nitroso-tris-chloroethylurea (NTCU) (Santa Cruz sc-212265) diluted in acetone applied twice weekly for 12 weeks. Controls received only acetone. Mice were monitored for a further 11 weeks and weighed twice weekly. On sacrifice, lungs were insuflatted with 4% paraformaldehyde/PBS and fixed overnight at 4°C before paraffin embedding.

### Histology and immunofluorescence

Haematoxylin and eosin staining was performed on an automated staining system (Tissue-Tek). Immunofluorescence and immunohistochemical staining were performed using standard protocols. Antigen retrieval methods, antibodies, reagents and equipment are detailed in the supplementary methods.

### Bioinformatic analyses

Analyses of the Human Lung Cell Atlas single-cell RNA sequencing (scRNAseq) dataset [22] were conducted as detailed in the supplementary methods. We assessed *LRIG1* expression in two datasets of human pre-invasive squamous cell lung cancer lesions obtained at bronchoscopy downloaded from the Gene Expression Omnibus. The first dataset, GSE33479, contained 122 samples of pre-invasive lesions, from normal epithelium to invasive cancer, profiled using Agilent microarrays [10]. The second, GSE94611 and GSE108082, contained laser-captured epithelial samples from 51 CIS lesions (progressive and regressive) [11]. Data were analysed in the R statistical environment (version 3.5.0; www.r-project.org) using Bioconductor version 3.7.

### Statistical analyses

Statistical analysis was performed on Prism 7 (GraphPad). Tests and sample sizes are indicated in figure legends; p<0.05 was considered to be statistically significant.

## Results

### LRIG1 is heterogeneously expressed in the airway epithelium

**FIGURE 1:**
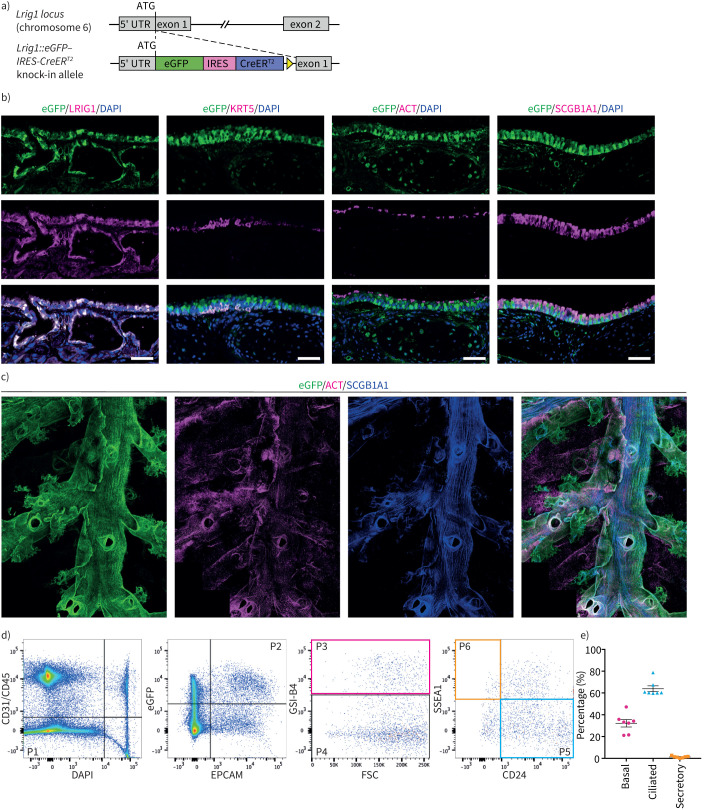
Expression of leucine-rich repeats and immunoglobulin-like domains 1 (*Lrig1*) in the murine airways. a) Schematic representation of wild-type and knock-in reporter *Lrig1* alleles. An enhanced green fluorescent protein (eGFP)-internal ribosome entry site (IRES)-CreER^T2^ cassette was targeted to the start codon of *Lrig1*, shifting the endogenous gene out of frame. b) Immunofluorescence images of tracheal sections from mice heterozygous for the knock-in reporter *Lrig1* allele demonstrate that expression of eGFP (green) recapitulates endogenous LRIG1 protein (magenta). Expression of eGFP is detected in different cell compartments of the airway epithelium, including KRT5^+^ basal cells, acetylated-tubulin (ACT^+^) ciliated cells and SCGB1A1^+^ secretory cells. Scale bars=50 μm. c) Whole-mount preparation of an adult mouse lung showing expression of eGFP in the lung airways. Antibody staining for SCGB1A1 and ACT was used for identification of club and ciliated cells, respectively. d) Immunophenotypic characterisation of tracheal epithelial cells expressing *Lrig1::eGFP* by flow cytometry. Live tracheal epithelial cells (P1) were identified by negative selection of cells expressing CD31, CD45 (endothelial cells and lymphocytes) and 4′,6-diamidino-2-phenylindole (DAPI), followed by positive selection of epithelial cell adhesion molecule (EPCAM)^+^eGFP^+^ cells (P2). *Griffonia simplicifolia* isolectin B4 (GSI-B4) labelling was used to distinguish basal cells (P3) within the EPCAM^+^eGFP^+^ population. Ciliated and secretory cells were selected within the GSI-B4-negative fraction by expression of CD24 (P5) and SSEA1 (P6), respectively. e) Distribution of cell types in the eGFP^+^ tracheal epithelium (mean±sem, n=7).

Using immunofluorescence, we have shown previously that LRIG1 is expressed in the murine upper airways [18]. To characterise its expression within the different cell subpopulations of the airway epithelium we used an *Lrig1* knock-in reporter mouse. In this model, a cassette encoding *eGFP-IRES-CreER^T2^* is inserted downstream of the *Lrig1* start codon (figure 1a) [23]. Enhanced green fluorescent protein (eGFP) expression occurs from the endogenous *Lrig1* promoter and results in a loss-of-function allele. As shown previously for the skin and intestine [23], antibody sta­ining confirmed expression of eGFP recapitulates endogenous LRIG1 in the upper airways (figure 1b).

In both murine skin and intestinal epithelia, LRIG1 localises to defined stem cell compartments where it regulates stem cell activity [24, 25]. In contrast, *Lrig1::eGFP* expression was evident throughout the upper airway epithelium. eGFP was detected in basal (KRT5^+^), ciliated (acetylated-tubulin (ACT^+^)) and, to a lesser extent, in club (SCGB1A1^+^) epithelial cells (figure 1b). There was no obvious enrichment of eGFP within the KRT5^+^ cell subpopulation of the submucosal glands, where a reservoir of stem cells that contribute to the regeneration of the surface epithelium after severe injury reside [26, 27] (figure 1b). Whole-mount immunostaining in the adult murine lung demonstrated *Lrig1::eGFP* expression extended throughout the bronchial tree, including the bronchi and bronchioles (figure 1c).

To determine the contribution of basal, ciliated and secretory cells to the cellular population expressing *Lrig1::eGFP* in upper airway epithelium we used flow cytometry. Tracheal epithelial cells were isolated from mice carrying one copy of the *Lrig1::eGFP* reporter allele. The immune and endothelial populations were eliminated through negative selection for CD45 and CD31, respectively. *Lrig1*^+^ epithelial cells were identified by dual expression of epithelial cell adhesion molecule (EPCAM) and eGFP. *Griffonia simplicifolia* isolectin B4 (GSI-B4), which selectively binds to a cell surface carbohydrate found on airway basal cells [28, 29], was used to identify the basal cell population. CD24 and SSEA1 expression were used to separate ciliated and secretory cells, respectively [30] (figure 1d). Basal cells constituted mean±sem 32.2±3.4% of the *Lrig1::eGFP*^+^ population, whereas ciliated and secretory cells made up the 63.9±2.7% and 1.3±0.3%, respectively (figure 1e). The distribution of cell types did not change significantly with selection of the brightest eGFP^+^ cells (supplementary figure S1), indicating that *Lrig1* expression is not enriched within the basal cell compartment. Only 50.8±4.1% of the total airway basal cell population was *Lrig1::eGFP*^+^.

### Murine and human basal airway epithelial cells expressing *Lrig1* have increased *in vitro* self-renewal capacity

**FIGURE 2:**
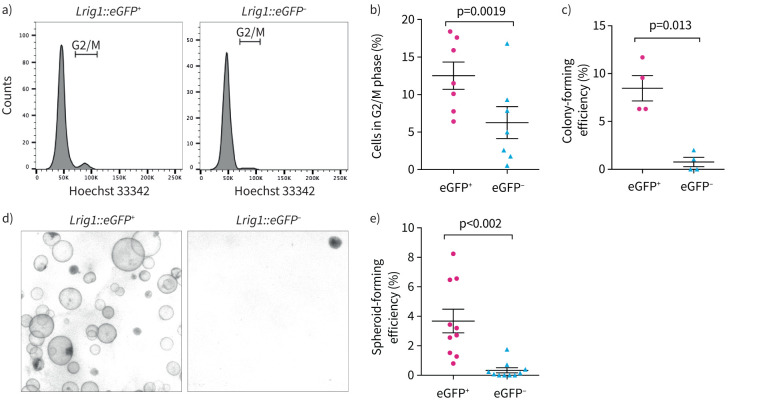
Mouse basal airway epithelial cells expressing leucine-rich repeats and immunoglobulin-like domains 1 (LRIG1) have increased *in vitro* self-renewal potential. a) epithelial cell adhesion molecule (EPCAM)^+^ *Griffonia simplicifolia* isolectin B4 (GSI-B4)^+^ basal murine airway epithelial cells from *Lrig1::eGFP* heterozygous mice were purified by flow cytometry from freshly dissociated tissue following depletion of the CD31^+^ and CD45^+^ populations. Hoechst 33342 staining was used to compare DNA content in the enhanced green fluorescent protein (eGFP)-positive and -negative fractions. b) The proportion of cells in G2/M phase is higher in eGFP^+^ basal cells compared to eGFP^–^ basal cells (mean±sem, t-test, n=7 biological replicates). c) Single eGFP^+^ or eGFP^–^ basal cells were sorted by flow-cytometry into individual wells of 96-well plates containing mitotically inactivated feeder cells. The number of cells giving rise to colonies containing >10 cells was assessed 10 days after plating (paired t-test, n=4 mice). d) and e) The ability of eGFP^+^ and eGFP^–^ basal cells to form spheroids was assessed following 14 days of culture in Matrigel (mean±sem, paired t-test, n=10 mice).

As only a subpopulation of airway basal epithelial cells expressed *Lrig1*, we investigated whether the properties of the *Lrig1*-expressing basal cells differ from the *Lrig1*-negative subpopulation. EPCAM^+^GSI-B4^+^ airway basal epithelial cells were isolated from murine tracheas heterozygous for the *Lrig1::eGFP* allele. Cell-cycle analysis revealed the *Lrig1::eGFP*^+^ basal cell subpopulation contained a higher proportion of cells in G2/M compared to the *Lrig1::eGFP^–^* fraction (t-test p=0.0019) (figure 2a and b). This agrees with previous skin and stomach findings where *Lrig1* expression identifies more proliferative stem/progenitor cell populations [21, 23, 24].

To determine whether *Lrig1*-expressing basal cells display increased self-renewal potential, we assessed their clonogenic potential in two-dimensional culture. Freshly isolated single *Lrig1::eGFP^+^* and *Lrig1::eGFP^–^* basal cells from mice heterozygous for the reporter allele were sorted into 96-well plates and assessed for colony-forming ability at day 10. Basal cells expressing *Lrig1::eGFP* formed significantly more clones than those not expressing *Lrig1* (t-test p=0.013) (figure 2c). When seeded into Matrigel, basal airway epithelial cells give rise to three-dimensional organoids called “tracheospheres” [31]. Under these conditions, *Lrig1::eGFP^+^* basal cells generated significantly more tracheospheres than *Lrig1::eGFP^–^* cells (t-test p=0.002) (figure 2d and e), indicating that *Lrig1*-expressing murine basal epithelial cells have higher *in vitro* propagation potential.

**FIGURE 3:**
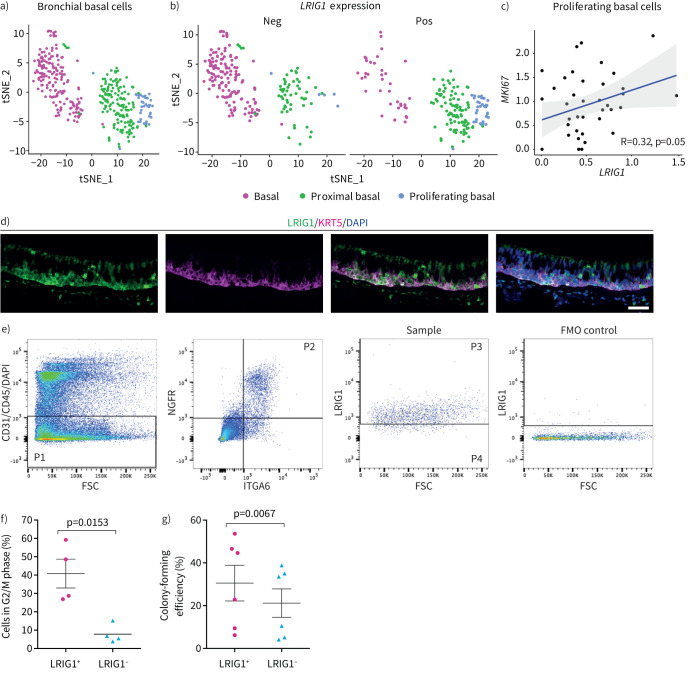
Expression of leucine-rich repeats and immunoglobulin-like domains 1 (LRIG1) in human basal airway epithelial cells is associated with increased proliferation. The single-cell RNA sequencing dataset from the Human Lung Cell Atlas was used to examine differences between bronchial basal cells with or without *LRIG1* expression. a) t-distributed stochastic neighbor embedding (t-SNE) dimensional reductions displaying the major basal cell clusters present in bronchial regions. b) Count of cells with (Pos) or without (Neg) *LRIG1* expression in each basal cell subpopulation. c) Scatter plot displaying the correlation between *LRIG1* and *MKI67* expression in proliferating basal cells from the Human Lung Cell Atlas. d) Immunofluorescence staining for LRIG1 and the basal cell marker KRT5 in the human airway epithelium. Scale bar=50 μm. e) Integrin α-6 (ITGA6)^+^ nerve growth factor receptor (NGFR)^+^ human basal cells (P2) were isolated from endobronchial brushings by flow cytometry and separated into LRIG1^+^ (P3) and LRIG1^–^ (P4) subpopulations. Fluorescence minus one (FMO) control is shown. f) Cell-cycle analysis on live cells using Hoechst 33342 indicates that the LRIG1^+^ human basal cell fraction contains an increased proportion of cells in G2/M (mean±sem, paired t-test, n=4 patients). g) The proportion of individual LRIG1^+^ and LRIG1^–^ human basal cells giving rise to colonies after 10 days of culture on mitotically inactivated feeder cells is shown (mean±sem, paired t-test, n=6 patients).

Next, we investigated whether LRIG1 expression identifies a more proliferative basal cell population within the human airway epithelium. Examination of publicly available single-cell transcriptomic data of the human airway epithelium (www.lungcellatlas.org) [32] showed that similarly to the mouse, *LRIG1* is heterogeneously expressed by basal, ciliated and club cells (supplementary figure S2). Using the scRNAseq dataset from the Human Lung Cell Atlas [22], we assessed *LRIG1* expression within the different clusters of basal cells present in the human bronchial epithelium. This revealed that *LRIG1* expression enriches for “proliferating basal cells”. The “basal cell” cluster, which is quiescent, was enriched in the *LRIG1*-negative fraction. Cells with a “proximal basal” signature were present in both fractions, but appeared more abundant in the *LRIG1*^+^ one (figure 3a and b and supplementary figure S3). Within the proliferating basal cell cluster, *LRIG1* levels correlated with those of *MKI67*, which codes for the proliferation marker Ki67 (Pearson correlation *R*=0.32, p=0.05) (figure 3c).

Expression of LRIG1 within the KRT5^+^ basal cell compartment of the normal human bronchial epithelium was confirmed by double immunofluorescence (figure 3d). Normal human basal cells were isolated by flow cytometry from endobronchial brushings obtained during autofluorescence bronchoscopy. Following elimination of CD45^+^, CD31^+^ and dead cells, co-expression of the cell-surface proteins integrin α-6 (ITGA6) and nerve growth factor receptor (NGFR), which are enriched in airway basal cells [31] (supplementary figure S4), was used to identify this cell population. Basal cells were then separated based on LRIG1 immunoreactivity (figure 3e). Live-cell cell-cycle analysis demonstrated an increased proportion of cells in G2/M in human basal cells expressing LRIG1 compared to the LRIG1-negative fraction (paired t-test p=0.015) (figure 3f). When colony-forming ability was assessed, LRIG1^+^ cells gave rise to colonies with a higher efficiency than their LRIG1^–^ counterparts (paired t-test p=0.0067) (figure 3g). Together, our results show that expression of LRIG1 within the basal cell compartment identifies a more proliferative cellular subpopulation with increased *in vitro* propagating potential in both murine and human airway epithelium.

LRIG1 functions as a negative regulator of EGFR signalling and thus LRIG1^+^ cells showing a greater proliferation seems paradoxical. Therefore, we hypothesised that LRIG1 expression is a checkpoint for proliferation employed by progenitor basal cells, but not needed in a low-proliferating population (LRIG1-negative). To test this, we knocked-down *LRIG1* in human basal cells and indeed noted enhanced cell population expansion (supplementary figure S5). Hence, LRIG1 marks a progenitor population of basal cells and functions as a checkpoint to proliferation.

### Progression of pre-invasive lung cancer lesions to invasive LUSC is associated with decreased *LRIG1* expression

**FIGURE 4:**
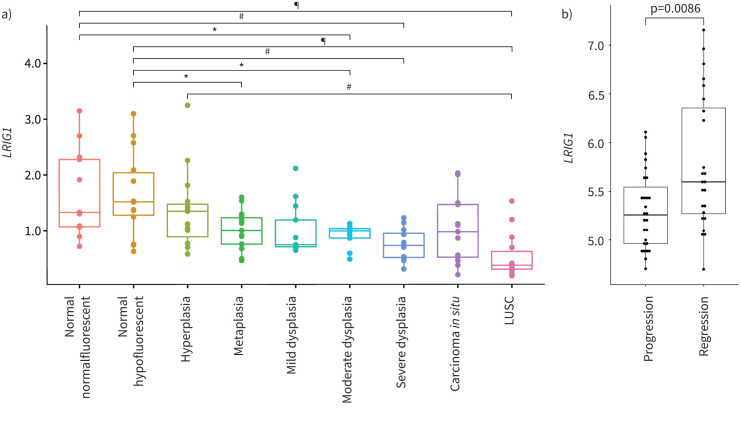
Expression of leucine-rich repeats and immunoglobulin-like domains 1 (*LRIG1*) during human lung squamous cell carcinoma (LUSC) progression. a) Comparison of *LRIG1* gene expression in 122 pre-invasive squamous cell lung cancer lesions across stages of progression to cancer, as defined by standard histological criteria. ANOVA p=3.7×10^−7^, Tukey's multiple comparisons test was used for between-group analysis. *: p<0.05, ^#^: p<0.005, ^¶^: p<0.00005. b) Analysis of *LRIG1* expression data from laser-captured carcinoma *in situ* lesions that either progressed to cancer (n=27) or spontaneously regressed (n=24), showing decreased expression of *LRIG1* in those that progressed to cancer. p=0.0086 (Wilcoxon rank sum test).

To better understand the role of LRIG1 in LUSC formation, we examined expression of *LRIG1* at different stages of human LUSC development. We analysed a published dataset including patient biopsies from normal bronchial tissue, six morphologically distinct grades of pre-invasive disease, ranging from hyperplasia to CIS, and LUSC [10]. This revealed decreased *LRIG1* expression in pre-invasive samples that varied from metaplasia to severe dysplasia, relative to normal tissue (p<0.05) (figure 4a). Further decline in *LRIG1* levels was observed in LUSC samples when compared to normal and hyperplastic tissue (p=2×10^−5^ and p=0.0026, respectively) (figure 4a). Additionally, assessment of *LRIG1* expression in a longitudinally characterised cohort of CIS lesions that either progressed to invasive LUSC or regressed [11] showed significantly lower *LRIG1* levels in the progressive group (p=0.0086) (figure 4b). Together, these data indicate that a decrease of *LRIG1* expression is associated with progressive severity of pre-invasive disease through to invasive lung cancer.

### Establishment of a lung squamous cell carcinoma murine model

**FIGURE 5:**
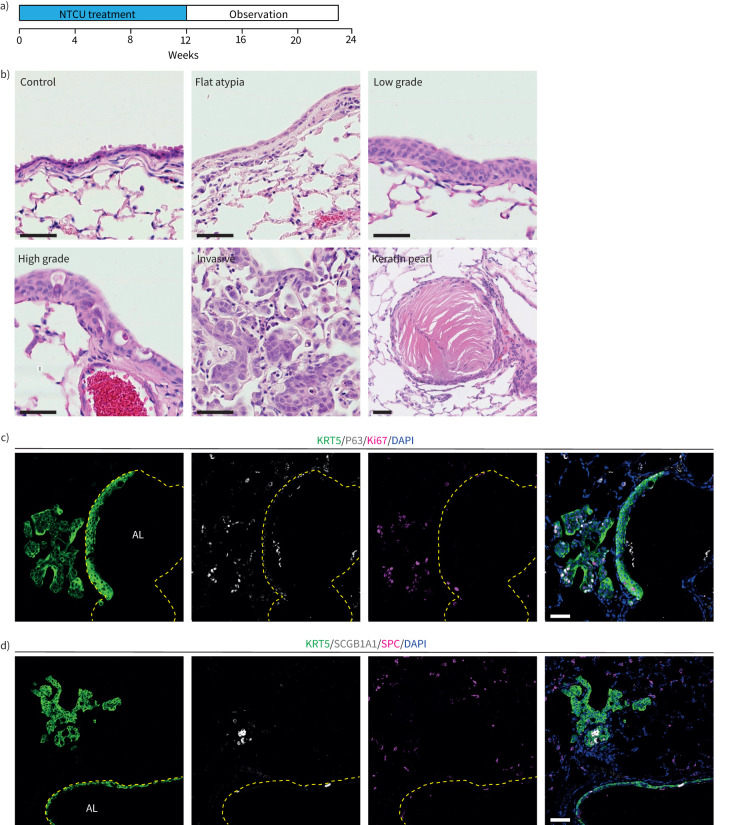
N-nitroso-tris-chloroethylurea (NTCU)-induced murine lung squamous cell carcinoma. a) NTCU administration protocol for the induction of murine lung squamous cell carcinoma. b) Representative images of NTCU-induced lung lesions stained with haematoxylin and eosin. Flat atypia with enlarged, flattened nuclei and increased nuclear-cytoplasmic ratio. Low-grade dysplasia with presence of multiple, ordered layers of epithelial cells and clear organisation from the basement membrane to the luminal surface. Note flattened nuclei adjacent to the lumen. High-grade dysplasia showing disordered layers of epithelial cells and multiple enlarged nuclei. Invasive squamous cell carcinoma lesion, beginning to fill the alveolar spaces. Keratin pearl, a characteristic feature of squamous cell carcinoma. c) Immunofluorescence staining showing expression of the lung squamous cell carcinoma markers P63 and KRT5, and the proliferation marker Ki67, in NTCU-induced lesions. d) Antibody staining demonstrates lack of immunoreactivity for the lung adenocarcinoma marker surfactant protein C within NTCU-induced lesions. Basement membrane (dashed lines) and airway lumen (AL) are indicated. Scale bars=50** **μm.

To investigate the consequences of decreased *LRIG1* levels during LUSC development, we used a chemically induced murine model of LUSC [33]. Topical application of NTCU to young-adult mice twice weekly for 12 weeks resulted in LUSC formation over the next 11 weeks (figure 5a). Histological analysis revealed that NTCU-induced lesions recapitulated the various stages of the human disease, from pre-invasive lesions to invasive LUSC (figure 5b). Pre-invasive lesions and tumours expressed the LUSC markers KRT5 and P63 and lacked expression of the lung adenocarcinoma (LUAD) marker surfactant protein C (SPC). Rare KRT5^+^ cells expressing low levels of SCGB1A1 were detected within NTCU-induced lesions (figure 5c and d).

### *Lrig1* loss of function leads to increased tumour size in a murine LUSC model

**FIGURE 6:**
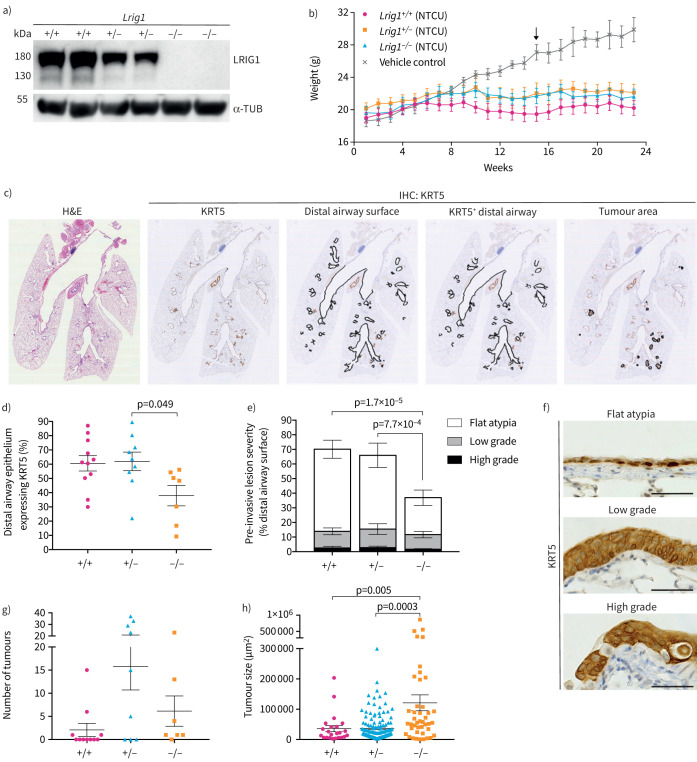
Effects of leucine-rich repeats and immunoglobulin-like domains 1 (*Lrig1*) loss-of-function on N-nitroso-tris-chloroethylurea (NTCU)-induced lung carcinogenesis. a) Immunoblot for LRIG1 on cerebellar tissue lysates from wild-type, *Lrig1*-heterozygous and *Lrig1*-null mice. Note that protein levels change in a gene-dosage dependent manner. α-tubulin is shown as a loading control. b) Weights of NTCU-treated and control animals (mean±sem, *Lrig1*^+/+^ n=11, *Lrig1*^+/–^ n=9, *Lrig1*^–/–^ n=7). Two-way ANOVA: time effect (p<0.0001) and time × treatment interaction (p<0.0001). Arrow indicates significant differences between all treatment groups and control arm. Tukey's multiple comparisons test, p<0.05. c) Transverse lung sections of NTCU-treated mice stained with haematoxylin and eosin and processed for KRT5 immunohistochemistry to identify pre-invasive airway lesions and invasive tumours. d) Proportion of lower airway epithelium displaying abnormal KRT5 expression in wild-type (+/+), *Lrig1*-heterozygous (+/−) and *Lrig1*-null (−/−) mice treated with NTCU (mean±sem, Kruskal–Wallis test, p=0.033, followed by Dunn's multiple comparisons test, p=0.049; n=7–11 mice per group). e) KRT5-expressing lesions were categorised as flat atypia, low-grade or high-grade. Distribution of pre-invasive lesions of different grades within lower airway (mean±sem). Two-way ANOVA: genotype effect (p=0.009) and lesion grade effect (p<1×10^−6^). The presence of flat atypia was statistically different among genotypes (Tukey's multiple comparisons test, p=7.7×10^−4^, p=1.7×10^−5^). f) Representative images of NTCU-induced pre-invasive lesions stained for KRT5. Scale bars=50 μm. g) Number of invasive tumours per mouse. Kruskal–Wallis indicated no significant differences among groups (n=7–11 mice per genotype). h) Size of invasive tumours in mice of different *Lrig1* genotypes (mean±sem). Kruskal–Wallis test, p=0.0002, followed by Dunn's multiple comparisons test, p=0.005, p=0.0003; *Lrig1*^+/+^ n=23, *Lrig1*^+/–^ n=142, *Lrig1*^–/–^ n=43 invasive lesions. IHC: immunohistochemistry.

The *Lrig1::eGFP-IRES-CreER^T2^* knock-in allele leads to *Lrig1* loss of function (figure 6a). To establish whether decreased *Lrig1* gene dosage impacts LUSC formation, we compared effects of NTCU in *Lrig1*-null, -heterozygous and wild-type mice. Animals were monitored for weight change and there was a marked separation between the treatment and control arms from week 15 (two-way ANOVA, p<0.05) (figure 6b).

KRT5^+^ basal cells are restricted to the upper airway murine epithelium [34]. Thus, expression of KRT5 beyond the trachea and the divisions of the right and left mainstem bronchi is abnormal. Therefore, we used KRT5 immunostaining to assess the presence of pre-invasive lesions in the bronchial tree following NTCU treatment. The proportion of total bronchial tree affected by pre-invasive disease in each mouse was calculated (figure 6c) and compared between *Lrig1*-null, -heterozygous and wild-type mice. *Lrig1* genotype had an effect on the extent of pre-invasive disease (Kruskal–Wallis test, p=0.033), with the *Lrig1*-null (*Lrig1^−/–^*) group displaying lower overall bronchial epithelial surface with abnormal KRT5 expression, when compared to *Lrig1* heterozygous (*Lrig1^+/–^*) mice (Dunn's multiple comparisons test, p=0.049) (figure 6d). Areas of pre-invasive disease were divided according to severity into flat atypia, low-grade dysplasia and high-grade dysplasia (table 1 and figure 6e and f). There were comparable proportions of both low-grade and high-grade lesions across groups (figure 6e). However, the presence of flat atypia, the earliest feature of pre-invasive disease, was significantly lower in the *Lrig1*-null animals when compared to both wild-type and *Lrig1^+/–^* mice (two-way ANOVA followed by Tukey's multiple comparisons tests, p<0.001) (figure 6e).

**TABLE 1:** Incidence of N-nitroso-tris-chloroethylurea-induced pre-invasive lesions and lung squamous cell carcinoma (LUSC) in leucine-rich repeats and immunoglobulin-like domains 1 (*Lrig1*)-deficient and control mice

	**Percentage incidence^#^**			
	**Flat atypia (n/N)**	**Low-grade (n/N)**	**High-grade (n/N)**	**LUSC (n/N)**
***Lrig1* genotype**				
+/+	100 (11/11)	100 (11/11)	90.90 (10/11)	36.36 (4/11)
+/−	100 (9/9)	100 (9/9)	88.89 (8/9)	66.67 (6/9)
−/−	100 (7/7)	100 (7/7)	85.71 (6/7)	85.71 (6/7)

^#^: number of animals with lesions/total number of animals.

Next, we assessed invasive tumours in the three groups. There was a trend towards increased incidence of LUSC as *Lrig1* gene dosage decreased (Chi-squared test for trend, p=0.03) (table 1). When we compared the frequency and size of tumours between animals with different *Lrig1* genotypes, *Lrig1* gene dosage had no significant effect on the number of tumours per individual (figure 6g). However, the absence of LRIG1 led to significantly larger tumours than those in mice with active LRIG1 expression (Kruskal–Wallis test, p=0.0002) (figure 6h). Larger tumours, alongside the lower frequency of flat atypia in the *Lrig1*-null lungs, suggests loss of *Lrig1* facilitates and/or accelerates pre-invasive disease progression into invasive LUSC.

**FIGURE 7:**
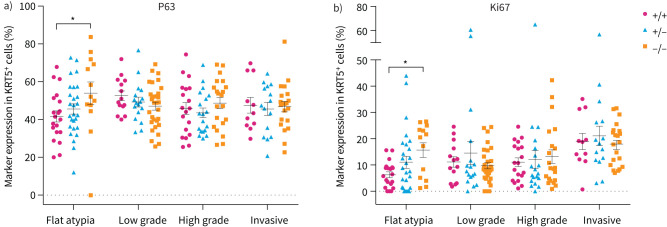
Characterisation of N-nitroso-tris-chloroethylurea (NTCU)-induced lesions. a) Expression of P63 in KRT5-expressing squamous airway lesions of different grades in wild-type, leucine-rich repeats and immunoglobulin-like domains 1 (*Lrig1*)-heterozygous and *Lrig1*-null mice (mean±sem). b) Ki67 immunoreactivity in KRT5-expressing squamous airway lesions of increasing grades in *Lrig1*^+/+^, *Lrig1*^+/–^ and *Lrig1*^–/–^ mice (mean±sem). For each lesion grade, the effect of *Lrig1* genotype on the fraction of KRT5^+^ cells expressing each marker was assessed. Kruskal–Wallis test, followed by Dunn's multiple comparisons test, *: p<0.05. For both a) and b) n=11–31 lesions/grade/genotype; KRT5^+^ cells: *Lrig1*^+/+^ n=6776, *Lrig1*^+/–^ n=10 206, *Lrig1*^–/–^ n=12 845.

To determine whether LRIG1 regulates LUSC progression by modulating cell fate or promoting proliferation, we investigated expression of P63 and Ki67 within the different spectrum of lesions. KRT5^+^ lesions were classified according to their severity and the fraction of cells expressing each marker (regardless of expression levels) were scored. The overall proportion of P63^+^ cells in NTCU-induced lung lesions was not significantly affected by *Lrig1* genotype or lesion grade (two-way ANOVA, p>0.05) (figure 7a). However, when comparisons were made only among lesions of the same grade, we found that flat atypia lesions contained a significantly higher proportion of P63^+^ cells in *Lrig1*-null mice than in their wild-type counterparts (Kruskal–Wallis test followed by Dunn's multiple comparison test, p=0.04). Assessment of the fraction of Ki67^+^ cells revealed differences between distinct lesion grades (two-way ANOVA, lesion grade effect, p=0.0007). When we evaluated genotype effects within lesions of the same grade, we found that in comparison to wild-type mice, mice lacking *Lrig1* also exhibited an increased fraction of Ki67^+^ cells in flat atypia lesions (Kruskal–Wallis test followed by Dunn's multiple comparison test, p=0.04) (figure 7b). This suggests that LRIG1 activity restrains LUSC formation by restricting progenitor cell proliferation in early pre-invasive disease.

## Discussion

Studies of the homeostatic upper airway epithelium in mouse and human highlight phenotypic and functional heterogeneity within the basal cell compartment. Current evidence indicates basal cells comprise a population of multipotent stem cells and committed progenitors [32, 35–37]. Here we find that *Lrig1* is heterogeneously expressed in the airway epithelium and its expression identifies a more proliferative subpopulation of basal cells with increased *in vitro* self-renewal potential. These findings are in line with previous studies in the epidermis and forestomach, where LRIG1 expression marks proliferative epithelial stem/progenitor cells, and in glandular gastric epithelial cells, where *Lrig1*-expressing cells have higher organoid-forming ability than their *Lrig1*-negative counterparts [21, 23]. This suggests a conserved role for LRIG1 in regulation of progenitor cell activity in distinct epithelia.

In contrast to the skin and intestine, where *Lrig1* is enriched in the stem cell compartment [24, 25], in the murine gastric epithelium *Lrig1* is expressed by both progenitor and differentiated cells [23, 38]. Similarly, we find that in the upper airways, expression of *Lrig1* is detected in basal, ciliated and secretory cells. In the mouse distal airways, scRNAseq analyses have shown that *Lrig1* is enriched in SCGB1A1^+^ club cells and expressed at moderate levels by SPC^+^SCGB1A1^+^ bronchioalveolar stem cells [39]. Analogous studies of the human airways show that *LRIG1* is heterogeneously expressed across the different epithelial cell subpopulations [32]. This raises the possibility that LRIG1 may exert functional roles in airway epithelial stem cells, lineage-restricted progenitors and differentiated cells. In future studies it would be important to determine how LRIG1 dysregulation affects these different populations using lineage-specific loss- or gain-of-function approaches.

Dysregulation of LRIG1 is seen across different tumour types [40]. Decreased *LRIG1* expression has been associated with poor prognosis in a range of epithelial tumours, including lung cancer [19, 20, 41]. We now show that downregulation of *LRIG1* occurs early during LUSC evolution, before development of high-grade lesions and invasive tumours. Primary human pre-invasive squamous lung cancer has not been grown in culture, limiting functional investigation of the molecular alterations occurring at this stage of LUSC development. Here we find that *LRIG1* knockdown enhances *in vitro* propagation of primary basal cells isolated from the human normal airway epithelium, supporting a potential regulatory role in pre-invasive disease development. Recently, air liquid interface cultures of immortalised human bronchial epithelial cells with loss of *TP53* and *SOX2* overexpression have been shown to recapitulate bronchial dysplasia features [42]. This model may be useful to assess the consequences of loss of *LRIG1* in pre-invasive squamous cancer in future studies.

In an NTCU-induced murine model of LUSC, we found a trend towards increased LUSC development as *Lrig1* gene dosage decreased. The absence of *Lrig1* led to decreased presence of flat atypia and formation of larger invasive tumours. A correlation between lower LRIG1 expression and increased tumour size has been identified in hepatocellular carcinoma [43]. Gain- and loss-of-function studies in cancer cell lines from lung, breast and liver indicate that LRIG1 modulates cancer cell proliferation [43–45]. Assessment of Ki67 expression in NTCU-induced lesions showed that *Lrig1* depletion leads to abnormally high proliferative rates in early flat atypia lesions, but not in dysplasia or invasive tumours. These observations suggest the larger tumours in the *Lrig1*-null lungs result from accelerated disease progression, rather than from increased cell proliferation in established tumours.

We have shown that LRIG1 exerts a tumour suppressive role in LUSC development. Therefore, *LRIG1* loss may be used as a biomarker of poor outcome of pre-invasive lesions and lead to a lower threshold for intervention. With the loss of the break of LRIG1 in EGFR signalling contributing to the development of pre-invasive disease, our work highlights a potential role for EGFR antagonists in LUSC prevention. Other data suggest a key role in the suppression of LUAD. As a negative regulator of EGFR signalling, high LRIG1 expression is associated with a dramatic 2.8-year improved survival in LUAD patients [20]. Decreased *LRIG1* transcript levels have been reported in LUAD cell lines, especially those with mutant EGFR [44]. Transfection of EGFR-mutant LUAD cells with *LRIG1* led to decreased proliferation, invasion and migratory potential [44].

To date, functional analyses of the role of LRIG1 in NSCLC have relied on *in vitro* studies and xenograft models using LUAD cell lines [18, 44]. Here, we have used a chemically induced model of LUSC that develops endogenous cancer lesions recapitulating the histopathological features of the human disease, including step-wise progression through pre-invasive stages. RNA sequencing analyses of NTCU-induced tumours indicate that their transcriptional landscape mimics human LUSC [46], supporting the relevance of this model for understanding LUSC progression. We show that using NTCU-induced carcinogenesis in the context of a transgenic murine model enables assessment of gene function during different stages of LUSC development and provides a system for validating candidate tumour suppressors, oncogenes and therapeutic targets for the prevention and treatment of LUSC.

## Supplementary material

10.1183/13993003.00816-2020.Supp1 **Please note:** supplementary material is not edited by the Editorial Office, and is uploaded as it has been supplied by the author.
Supplementary material ERJ-00816-2020.Supplement

## Shareable PDF

10.1183/13993003.00816-2020.Shareable1 This one-page PDF can be shared freely online.
Shareable PDF ERJ-00816-2020.Shareable

## References

[1] Bray F, Ferlay J, Soerjomataram I, et al. Global cancer statistics 2018: GLOBOCAN estimates of incidence and mortality worldwide for 36 cancers in 185 countries. CA Cancer J Clin 2018; 68: 394–424. doi:10.3322/caac.21492 30207593

[2] Cheng TY, Cramb SM, Baade PD, et al. The international epidemiology of lung cancer: latest trends, disparities, and tumor characteristics. J Thorac Oncol 2016; 11: 1653–1671. doi:10.1016/j.jtho.2016.05.021 27364315 PMC5512876

[3] Youlden DR, Cramb SM, Baade PD. The International Epidemiology of Lung Cancer: geographical distribution and secular trends. J Thorac Oncol 2008; 3: 819–831. doi:10.1097/JTO.0b013e31818020eb 18670299

[4] Herbst RS, Morgensztern D, Boshoff C. The biology and management of non-small cell lung cancer. Nature 2018; 553: 446–454. doi:10.1038/nature25183 29364287

[5] Auerbach O, Stout AP, Hammond EC, et al. Changes in bronchial epithelium in relation to cigarette smoking and in relation to lung cancer. N Engl J Med 1961; 265: 253–267. doi:10.1056/NEJM196108102650601 13685078

[6] Nicholson AG, Perry LJ, Cury PM, et al. Reproducibility of the WHO/IASLC grading system for pre-invasive squamous lesions of the bronchus: a study of inter-observer and intra-observer variation. Histopathology 2001; 38: 202–208. doi:10.1046/j.1365-2559.2001.01078.x 11260299

[7] de Groot P, Munden RF. Lung cancer epidemiology, risk factors, and prevention. Radiol Clin North Am 2012; 50: 863–876. doi:10.1016/j.rcl.2012.06.006 22974775

[8] Yoshida K, Gowers KHC, Lee-Six H, et al. Tobacco smoking and somatic mutations in human bronchial epithelium. Nature 2020; 578: 266–272. doi:10.1038/s41586-020-1961-1 31996850 PMC7021511

[9] Beane JE, Mazzilli SA, Campbell JD, et al. Molecular subtyping reveals immune alterations associated with progression of bronchial premalignant lesions. Nat Commun 2019; 10: 1856. doi:10.1038/s41467-019-09834-2 31015447 PMC6478943

[10] Mascaux C, Angelova M, Vasaturo A, et al. Immune evasion before tumour invasion in early lung squamous carcinogenesis. Nature 2019; 571: 570–575. doi:10.1038/s41586-019-1330-0 31243362

[11] Teixeira VH, Pipinikas CP, Pennycuick A, et al. Deciphering the genomic, epigenomic, and transcriptomic landscapes of pre-invasive lung cancer lesions. Nat Med 2019; 25: 517–525. doi:10.1038/s41591-018-0323-0 30664780 PMC7614970

[12] van Boerdonk RA, Sutedja TG, Snijders PJ, et al. DNA copy number alterations in endobronchial squamous metaplastic lesions predict lung cancer. Am J Respir Crit Care Med 2011; 184: 948–956. doi:10.1164/rccm.201102-0218OC 21799074

[13] Pennycuick A, Teixeira VH, AbdulJabbar K, et al. Immune surveillance in clinical regression of preinvasive squamous cell lung cancer. Cancer Discov 2020; 10: 1489–1499. doi:10.1158/2159-8290.CD-19-1366 32690541 PMC7611527

[14] Laederich MB, Funes-Duran M, Yen L, et al. The leucine-rich repeat protein LRIG1 is a negative regulator of ErbB family receptor tyrosine kinases. J Biol Chem 2004; 279: 47050–47056. doi:10.1074/jbc.M409703200 15345710

[15] Gur G, Rubin C, Katz M, et al. LRIG1 restricts growth factor signaling by enhancing receptor ubiquitylation and degradation. EMBO J 2004; 23: 3270–3281. doi:10.1038/sj.emboj.7600342 15282549 PMC514515

[16] Viegas-Péquignot E, Flury-Hérard A, De Cremoux H, et al. Recurrent chromosome aberrations in human lung squamous cell carcinomas. Cancer Genet Cytogenet 1990; 49: 37–49. doi:10.1016/0165-4608(90)90162-4 2397472

[17] Sundaresan V, Ganly P, Hasleton P, et al. p53 and chromosome 3 abnormalities, characteristic of malignant lung tumours, are detectable in preinvasive lesions of the bronchus. Oncogene 1992; 7: 1989–1997. doi:10.1016/0169-5002(93)90371-4 1408139

[18] Lu L, Teixeira VH, Yuan Z, et al. LRIG1 regulates cadherin-dependent contact inhibition directing epithelial homeostasis and pre-invasive squamous cell carcinoma development. J Pathol 2013; 229: 608–620. doi:10.1002/path.4148 23208928 PMC3806036

[19] An Y, Zhao Z, Ou P, et al. Expression of LRIG1 is associated with good prognosis for human non-small cell lung cancer. Medicine 2015; 94: e2081. doi:10.1097/MD.0000000000002081 26632716 PMC5058985

[20] Kvarnbrink S, Karlsson T, Edlund K, et al. LRIG1 is a prognostic biomarker in non-small cell lung cancer. Acta Oncol 2015; 54: 1113–1119. doi:10.3109/0284186X.2015.1021427 25813475

[21] Page ME, Lombard P, Ng F, et al. The epidermis comprises autonomous compartments maintained by distinct stem cell populations. Cell Stem Cell 2013; 13: 471–482. doi:10.1016/j.stem.2013.07.010 23954751 PMC3793873

[22] Travaglini KJ, Nabhan AN, Penland L, et al. A molecular cell atlas of the human lung from single-cell RNA sequencing. Nature 2020; 587: 619–625. doi:10.1038/s41586-020-2922-4 33208946 PMC7704697

[23] Schweiger PJ, Clement DL, Page ME, et al. Lrig1 marks a population of gastric epithelial cells capable of long-term tissue maintenance and growth *in vitro*. Sci Rep 2018; 8: 15255. doi:10.1038/s41598-018-33578-6 30323305 PMC6189208

[24] Wong VW, Stange DE, Page ME, et al. Lrig1 controls intestinal stem-cell homeostasis by negative regulation of ErbB signalling. Nat Cell Biol 2012; 14: 401–408. doi:10.1038/ncb2464 22388892 PMC3378643

[25] Jensen KB, Collins CA, Nascimento E, et al. Lrig1 expression defines a distinct multipotent stem cell population in mammalian epidermis. Cell Stem Cell 2009; 4: 427–439. doi:10.1016/j.stem.2009.04.014 19427292 PMC2698066

[26] Tata A, Kobayashi Y, Chow RD, et al. Myoepithelial cells of submucosal glands can function as reserve stem cells to regenerate airways after injury. Cell Stem Cell 2018; 22: 668–683. doi:10.1016/j.stem.2018.03.018 29656943 PMC5935539

[27] Lynch TJ, Anderson PJ, Rotti PG, et al. Submucosal gland myoepithelial cells are reserve stem cells that can regenerate mouse tracheal epithelium. Cell Stem Cell 2018; 22: 653–667. doi:10.1016/j.stem.2018.03.017 29656941 PMC5935589

[28] Schoch KG, Lori A, Burns KA, et al. A subset of mouse tracheal epithelial basal cells generates large colonies *in vitro*. Am J Physiol Lung Cell Mol Physiol 2004; 286: L631–L642. doi:10.1152/ajplung.00112.2003 12959927

[29] Shimizu T, Nettesheim P, Mahler JF, et al. Cell type-specific lectin staining of the tracheobronchial epithelium of the rat: quantitative studies with *Griffonia simplicifolia* I isolectin B4. J Histochem Cytochem 1991; 39: 7–14. doi:10.1177/39.1.1701188 1701188

[30] Tata PR, Mou H, Pardo-Saganta A, et al. Dedifferentiation of committed epithelial cells into stem cells *in vivo*. Nature 2013; 503: 218–223. doi:10.1038/nature12777 24196716 PMC4035230

[31] Rock JR, Onaitis MW, Rawlins EL, et al. Basal cells as stem cells of the mouse trachea and human airway epithelium. Proc Natl Acad Sci USA 2009; 106: 12771–12775. doi:10.1073/pnas.0906850106 19625615 PMC2714281

[32] Vieira Braga FA, Kar G, Berg M, et al. A cellular census of human lungs identifies novel cell states in health and in asthma. Nat Med 2019; 25: 1153–1163. doi:10.1038/s41591-019-0468-5 31209336

[33] Wang Y, Zhang Z, Yan Y, et al. A chemically induced model for squamous cell carcinoma of the lung in mice: histopathology and strain susceptibility. Cancer Res 2004; 64: 1647–1654. doi:10.1158/0008-5472.CAN-03-3273 14996723

[34] Rock JR, Randell SH, Hogan BL. Airway basal stem cells: a perspective on their roles in epithelial homeostasis and remodeling. Dis Model Mech 2010; 3: 545–556. doi:10.1242/dmm.006031 20699479 PMC2931533

[35] Pardo-Saganta A, Law BM, Tata PR, et al. Injury induces direct lineage segregation of functionally distinct airway basal stem/progenitor cell subpopulations. Cell Stem Cell 2015; 16: 184–197. doi:10.1016/j.stem.2015.01.002 25658372 PMC4334442

[36] Watson JK, Rulands S, Wilkinson AC, et al. Clonal dynamics reveal two distinct populations of basal cells in slow-turnover airway epithelium. Cell Rep 2015; 12: 90–101. doi:10.1016/j.celrep.2015.06.011 26119728 PMC4518462

[37] Plasschaert LW, Žilionis R, Choo-Wing R, et al. A single-cell atlas of the airway epithelium reveals the CFTR-rich pulmonary ionocyte. Nature 2018; 560: 377–381. doi:10.1038/s41586-018-0394-6 30069046 PMC6108322

[38] Choi E, Lantz TL, Vlacich G, et al. Lrig1+ gastric isthmal progenitor cells restore normal gastric lineage cells during damage recovery in adult mouse stomach. Gut 2018; 67: 1595–1605. doi:10.1136/gutjnl-2017-313874 28814482 PMC5815959

[39] Liu Q, Liu K, Cui G, et al. Lung regeneration by multipotent stem cells residing at the bronchioalveolar-duct junction. Nat Genet 2019; 51: 728–738. doi:10.1038/s41588-019-0346-6 30778223

[40] Lindquist D, Kvarnbrink S, Henriksson R, et al. LRIG and cancer prognosis. Acta Oncol 2014; 53: 1135–1142. doi:10.3109/0284186X.2014.953258 25180912 PMC4438349

[41] Rouam S, Moreau T, Broet P. Identifying common prognostic factors in genomic cancer studies: a novel index for censored outcomes. BMC Bioinformatics 2010; 11: 150. doi:10.1186/1471-2105-11-150 20334636 PMC2863163

[42] Correia LL, Johnson JA, McErlean P, et al. SOX2 drives bronchial dysplasia in a novel organotypic model of early human squamous lung cancer. Am J Respir Crit Care Med 2017; 195: 1494–1508. doi:10.1164/rccm.201510-2084OC 28199128 PMC5470746

[43] Yang B, Dai C, Tan R, et al. Lrig1 is a positive prognostic marker in hepatocellular carcinoma. Onco Targets Ther 2016; 9: 7071–7079. doi:10.2147/OTT.S112534 27895499 PMC5117876

[44] Torigoe H, Yamamoto H, Sakaguchi M, et al. Tumor-suppressive effect of LRIG1, a negative regulator of ErbB, in non-small cell lung cancer harboring mutant EGFR. Carcinogenesis 2018; 39: 719–727. doi:10.1093/carcin/bgy044 29546323

[45] Miller JK, Shattuck DL, Ingalla EQ, et al. Suppression of the negative regulator LRIG1 contributes to ErbB2 overexpression in breast cancer. Cancer Res 2008; 68: 8286–8294. doi:10.1158/0008-5472.CAN-07-6316 18922900 PMC2597648

[46] Riolobos L, Gad EA, Treuting PM, et al. The effect of mouse strain, sex, and carcinogen dose on toxicity and the development of lung dysplasia and squamous cell carcinomas in mice. Cancer Prev Res 2019; 12: 507–516. doi:10.1158/1940-6207.CAPR-18-0442 PMC7687913 31101634

