# Supplementary material for: Lrig1 expression identifies airway basal cells with high proliferative capacity and restricts lung squamous cell carcinoma growth
Source: Eur Respir J. 2022 Mar 31;59(3):2000816. doi: 10.1183/13993003.00816-2020 (PMC8968013; doi:10.1183/13993003.00816-2020)
Supplement: Supplementary file 1 [file ERJ-00816-2020.Supplement.pdf]

***Lrig1* expression identifies airway basal cells with high proliferative capacity and restricts lung squamous cell carcinoma growth**

Laura Succony, Sandra Gómez-López, Adam Pennycuick, Ahmed S. N. Alhendi, Derek Davies, Sarah E. Clarke, Kate H.C. Gowers, Nicholas A. Wright, Kim B. Jensen, Sam M. Janes

Online Supplementary Material

SUPPLEMENTARY METHODS

Murine line background

The C57BL/6 *Lrig1::eGFP-IRES-CreERT*^{2} murine line was backcrossed twice to FVB/N and maintained in a mixed C57BL/6 and FVB/N background. The choice of mouse background was influenced by strain-specific differences to both the effects of NTCU-induced carcinogenesis and the loss of *Lrig1*.

Differences in the development of pre-invasive lesions and invasive LUSC across 8 mouse strains following the application of the carcinogen NTCU for 8 months have been reported in the literature [1]. C57BL/6 mice were among the strains with lowest susceptibility to NTCU, presenting only pre-invasive lesions. FVB/N displayed intermediate susceptibility to NTCU, and have been shown to be most sensitive in other lung carcinogenesis models [2]. Under shorter term NTCU application paradigms, mice with higher NTCU susceptibility presented high mortality rates [3]. To assess development of squamous cell carcinomas with medium-period NTCU treatments, the FVB/N strain was selected with the aim of recapitulating the full spectrum of disease, without leading to excess mortality.

The selection of strain was also influenced by the strain-specific effects of loss of *Lrig1*. In an outbred mouse model, loss of *Lrig1* was shown to result in skin hyperplasia [4]. However, *Lrig1* loss in a pure FVB/N background resulted in intestinal hyperplasia so severe that mice needed to be sacrificed around postnatal day 10 [5]. This would have prevented carcinogenesis studies on this strain.

Isolation of murine tissue & cells

Animals were sacrificed by overdose of sodium pentobarbital and tissues placed either into serum free DMEM (Gibco 41966) for epithelial cell isolation, 4% paraformaldehyde (PFA)/PBS for histology or ice-cold NP40 lysis buffer (50 mM Tris-HCl pH 8.0, 150 mM NaCl, 1% IGEPAL, 1X Halt Protease and Phosphatase inhibitor cocktail (Thermo Scientific 1861281)) in preparation for immunoblotting.

Murine tracheal epithelial cells were isolated as described [6]. Additionally, the discarded tracheal scaffold was digested 30 min in 0.25% collagenase, filtered through a 40 µm cell strainer and added to the cell suspension.

Isolation of human bronchial epithelial cells

Two bronchial brushings were collected from a normal area of bronchial mucosa into ice-cold 15 mL conical tubes containing collection media (αMEM supplemented with penicillin/streptomycin and amphotericin B). Brushes were vortexed vigorously to release the cells, with the brushes remaining inside the collection tube, and then centrifuged at 300 x *g* for 5 min. The cells were resuspended in red cell lysis buffer (Sigma R7757) and incubated at room temperature for three minutes. Cells were resuspended in 10% FBS, 1% BSA in PBS for blocking in preparation for flow cytometry staining.

For KRT5 expression analyses, proximal airway lobectomy specimens were digested with dispase and trypsin to obtain a single cell suspension. Following cell surface staining, cells were fixed in BD CellFIX (BD Biosciences 340181) for 15 min at room temperature and permeabilised with saponin for 15 min at 4°C. Cells were then incubated with Alexa Fluor 488-conjugated anti-KRT5 antibody (Abcam ab193894) for 15 min at 4°C. Alternatively, live ITGA6^{+}NGFR^{+} cells were purified, allowed to attached overnight onto collagen-coated chamber slides at 37°C and processed for immunofluorescence.

Colony forming assays

Cells were cultured at 37°C in 5% CO_2. Mitotically inactivated 3T3-J2 feeder layers were seeded at 20000 cells/cm^2 onto 96-well plates pre-coated with 0.05 mg/mL collagen type 1 (BD 354263) and left to adhere. The J2 media (DMEM supplemented penicillin/streptomycin (Gibco 15070) and 9% bovine serum (Gibco 26170)) was then changed to Murine Tracheal Epithelial Culture (MTEC) (for murine cells) or a 1:1 mixture of fresh FMED and J2/human bronchial epithelial cells (HBEC)-conditioned media (for human cells). MTEC consists of DMEM and F12 (Gibco 21765) in a 1:1 ratio supplemented with penicillin/streptomycin, 15 mM HEPES, 3.6 mM Na_2CO_3, 10 µg/mL insulin (Sigma I6634), 5 µg/mL transferrin (Sigma T1147), 0.1 µg/mL cholera toxin (Sigma C3012), 25 ng/mL EGF (BD 354001), 30 µg/mL bovine pituitary extract

(ThermoFisher 13028014), 5% foetal bovine serum (FBS, Gibco 10270) and freshly added 0.01 μM retinoic acid (Sigma R2625). FMED consists of DMEM and F12 in a 3:1 ratio supplemented 7.5% FBS, penicillin/streptomycin, 5 μM Y-27632 (ROCK inhibitor, Cambridge Bioscience Y1000), 25 ng/mL hydrocortisone (Sigma HO888), 0.125 ng/mL human recombinant EGF (Sino Biological 10605), 5 μg/mL insulin, 0.1 nM cholera toxin, 250 ng/mL amphotericin B (Fisher Scientific 10746254) and 10 μg/mL gentamicin (Gibco 15710). Single basal epithelial cells were sorted directly into each well. MTEC or FMED/conditioned media was changed 3 times a week. Human and murine cultures were fixed with 4% PFA at day 10 or 14, respectively, and stained with crystal violet for counting.

Tracheosphere assay

Flow cytometry-sorted murine basal epithelial cells were plated at 1250 cells/well in Matrigel (BD Biosciences 354230) and cultured in MTEC media. After 7 days, the media was changed to differentiation media, which is based on MTEC, except that contains a lower concentration of EGF (5 ng/mL) and cholera toxin (0.025 μg/mL), and includes 1 mg/mL bovine serum albumin (BSA). At day 14, spheres were counted, and imaged.

***LRIG1* knockdown in human bronchial epithelial cells**

Lentiviral vectors (pGIPZ) expressing green fluorescent protein (GFP) along with an shRNA against *LRIG1* (Open Biosystems V2LHS_229246) or a control non-silencing construct were used. Recombinant lentiviruses were produced by co-transfection of 293T cells with each pGIPZ vector together with packaging plasmids pCMVR8.74 and pMD2.G (Addgene plasmids #22036 and #12259) using the DNA transfection reagent jetPEI (Source Bioscience UK Ltd). Lentiviruses were concentrated by ultracentrifugation and titrated in 293T cells using GFP expression as readout. Human bronchial epithelial cells were expanded on 3T3-J2 feeder layers in FMED, as previously reported [7], and transduced at an multiplicity of infection (MOI) of 3 in the presence of 4 μg/mL polybrene (Sigma).

XTT proliferation assay

7,500 cells/well were plated into 96-well plates in complete BEGM (Lonza CC-2540B). Cells were left to adhere overnight, the media was then changed and XTT (Applichem

A8088) added at each time point according to the manufacturers' instructions. After 4h incubation, the absorbance was measured using a Titertec Multiscan MCC/340 plate reader (Labsystems, Turku, Finland) at wavelengths of 490 and 630 nm. The 630 nm measurement was subtracted from the 490 nm to obtain the absorbance reading. Percentage change was expressed as a proportion of the initial reading.

Immunoblotting

Dissected cerebella from 6-8-week-old mice were lysed in NP40 buffer. Human bronchial epithelial cells were lysed in RIPA buffer (Sigma R0278). Protein concentration of cleared supernatants was determined by BCA assays (Pierce Thermo Scientific 23225). Equivalent amounts of protein were resolved in 4-12% Bis-Tris gels and transferred onto nitrocellulose membranes. Membranes were blocked and incubated with primary antibodies (Supplementary Table 2) in 5% BSA/TBST overnight at 4°C. HRP-conjugated secondary antibodies were added and immunoblots analysed using Luminata Western HRP Chemiluminescence Reagent (Millipore WBLUR0500) on the ImageQuant LAS 4000 system (GE Healthcare).

Histology and immunofluorescence

Paraffin-embedded samples were sectioned at 4 μm. Heat-induced antigen retrieval was performed in 10 mM Sodium Citrate pH 6.0 in a microwave oven. This step was omitted when staining for GFP, in which case samples were permeabilised with 0.2% Triton X/PBS for 10 min before blocking. Fresh-frozen OCT-embedded human samples were sectioned, left to dry, fixed 10 min in 4%PFA and washed in PBS before incubating with blocking solution. For whole-mount immunofluorescence of mouse airways, the airways of the left lung lobe were microdissected under a stereomicroscope (Leica S9i) to expose the epithelium prior to antibody staining. Cells on collagen-coated chambers slides were fixed with 4%PFA for 10 min and washed with PBS before immunostaining. Primary antibodies were: GFP (Abcam ab13970), KRT5 (Abcam ab53121, BioLegend 905501 and 905901), human LRIG1 (R&D MAB7498), acetylated-tubulin (Sigma T6793), anti-SCGB1A1 (kind gift from Barry Stripp and Santa Cruz sc-9772), p63 (Abcam ab53039 and ab735, Cell Signaling Technology 13109), and Ki67 (eBioscience 14-5698). Secondary antibodies were conjugated to Alexa Fluor dyes (Life Technologies or Jackson ImmunoResearch) or streptavidin-HRP (DAKO). Fluorescently-labelled sections were mounted in Immu-

Mount (Thermo Scientific 9990402) or Fluoromount G (SouthernBiotech 01000-01). Whole-mount preparations were mounted in RapiClear 1.52 (SunJin Lab RC152001). Confocal images were obtained using a Zeiss LSM700 or an LSM880; epifluorescence images were acquired on a Leica DMi8 microscope. Image analyses were done using Fiji.

NTCU model analysis

Lungs from NTCU-treated and control groups were sectioned transversely until the trachea and main bronchi were visualised. Two 4 μm sections were cut, followed by two further sections 200 μm from the first. The first of the two sections were stained with haematoxylin and eosin (H&E) and the second immunostained for KRT5. Stained sections were imaged on a Nanozoomer (Hamamatsu). The NDP.view 2 software (Hamamatsu) was used to trace around the outline of the entire visible bronchial tree beyond the cartilage containing trachea and the beginning of right and left main bronchi (Fig. 5c). Any KRT5 staining distal to the origin of the right and left main bronchi was also drawn around. The KRT5 abnormal areas were categorised into each type of lesion: high-grade, low-grade or flat atypia. The percentage of bronchial tree affected by all pre-invasive lesions, together with the percentage of bronchial tree affected by each grade of lesion was calculated. A mean across two sections of bronchial tree was determined. Tumour size was determined by drawing around the circumference of each invasive area within a section. Lesions were counted as discrete if greater than 200 μm apart. Lesions across the two sections, 200 μm apart were included.

Analysis of the Human Lung Cell Atlas scRNAseq dataset

A scRNAseq dataset including ~75000 cells from three lung locations (alveolar, bronchiole and bronchi) was obtained from the Human Lung Cell Atlas (https://hlca.ds.czbiohub.org/) [8]. Using the cell type annotations provided in metadata, the single-cell RNA data from droplet 10X Chromium (10X) was filtered for normal human basal cells from epithelial tissue collected from the bronchi. The number of basal cells after filtering was 367. Genes enriched in each basal cell cluster have been described by Travaglini and colleagues [8]. The proliferative signature was defined by the expression of *PBK*, *BIRC5*, *MKI67*, *UBE2C*, *TOP2A*, *TK1*, *AURKB*, *CDKN3*, *CENPF*, *CDK1* and *ZWINT*. Analyses including QC metric, normalisation,

scaling and gene expression were performed using the R package Seurat v 4.0 [9]. Principal component analysis (PCA) dimensionality reduction was performed using the highly variable genes as input. The PCs were used to calculate t-distributed stochastic neighbour embedding (t-SNE). The number of PCs used for t-SNE was dataset-dependent and estimated by the elbow of a PCA scree plot. The average gene expression of selected genes and cell counts were calculated in each basal annotation after separating them based on *LRIG1* gene expression. The correlation between *LRIG1* and *MKI67* expressions in proliferating basal cells was preformed using Pearson correlation in R software.

Supplementary Table S1. Flow cytometry reagents

Reagent	Supplier	Code
Anti-mouse CD31	BioLegend	102407 and 102417
Anti-mouse CD45	BioLegend	103105
Anti-mouse EpCAM	BD Biosciences	563134
Isolectin GS-IB4 biotin conjugate	Invitrogen	I21414
Qdot 605 streptavidin conjugate	Invitrogen	Q10101MP
Anti-mouse CD24	BioLegend	101821
Anti-mouse SSEA1	eBioscience	46-8813-41
Anti-CD49f (ITGA6)	eBioscience	17-0495-80
Anti-human CD31	BioLegend	303106 and 303124
Anti-human CD45	BioLegend	368510 and 304032
Anti-human LRIG1	R&D Systems	FAB7498P
Anti-human NGFR	BioLegend	345110

Supplementary Table S2. Antibodies for immunoblotting

Antigen	Host	Supplier	Code	Concentration
mouse LRIG1	Rabbit	Abcam	Ab36707	1 µg/ml
human LRIG1	Rabbit	Cell Signaling Technology	#12752	1:1000
α-tubulin	Rabbit	Cell Signaling Technology	#9099	1:1000
EGFR	Rabbit	Cell Signaling Technology	#4267	1:1000
phospho-EGFR	Rabbit	Cell Signaling Technology	#2234	1:1000
ERK 1/2	Rabbit	Cell Signaling Technology	#9102	1:1000
phospho-ERK 1/2	Rabbit	Cell Signaling Technology	#9101	1:1000

SUPPLEMENTARY FIGURES

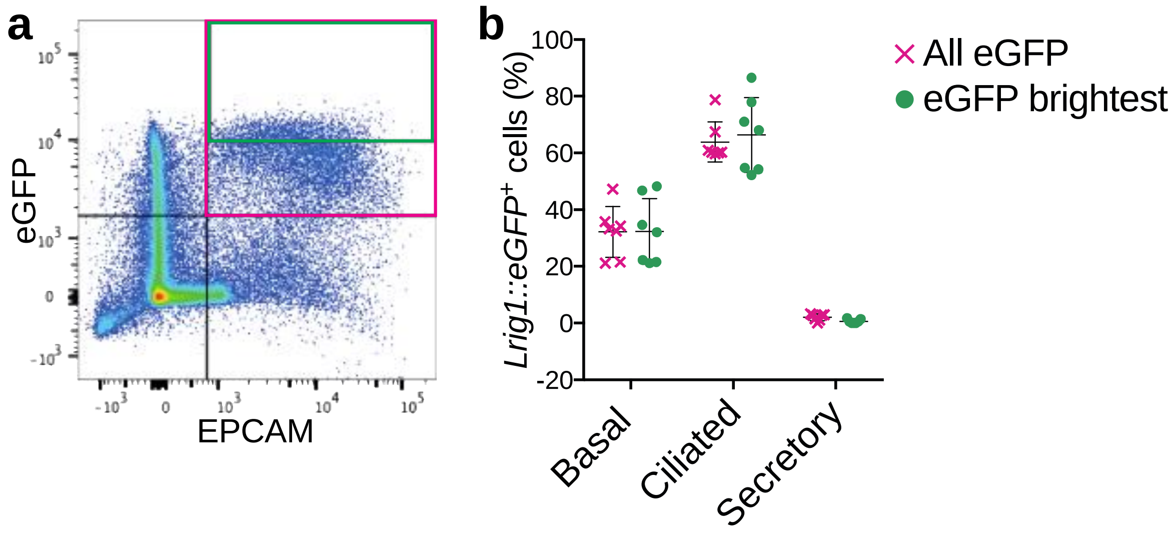

Supplementary Figure S1. Expression of *Lrig1::eGFP* in the murine upper airways. Immunophenotypic characterisation of tracheal epithelial cells expressing *Lrig1::eGFP* by flow cytometry. Live tracheal epithelial cells were identified by negative selection of cells expressing CD31, CD45 and DAPI, followed by positive selection of EPCAM$^+$eGFP$^+$ cells. **(a)** Flow cytometry plot showing total EPCAM$^+$eGFP$^+$ cells (magenta box) and the brightest eGFP$^+$ fraction (green box). **(b)** The distribution of GSI-B4$^+$ basal cells, ciliated CD24$^+$ cells and SSEA1$^+$ secretory cells were compared in the EPCAM$^+$eGFP$^+$ populations indicated in a (mean±SEM, *n*=7).

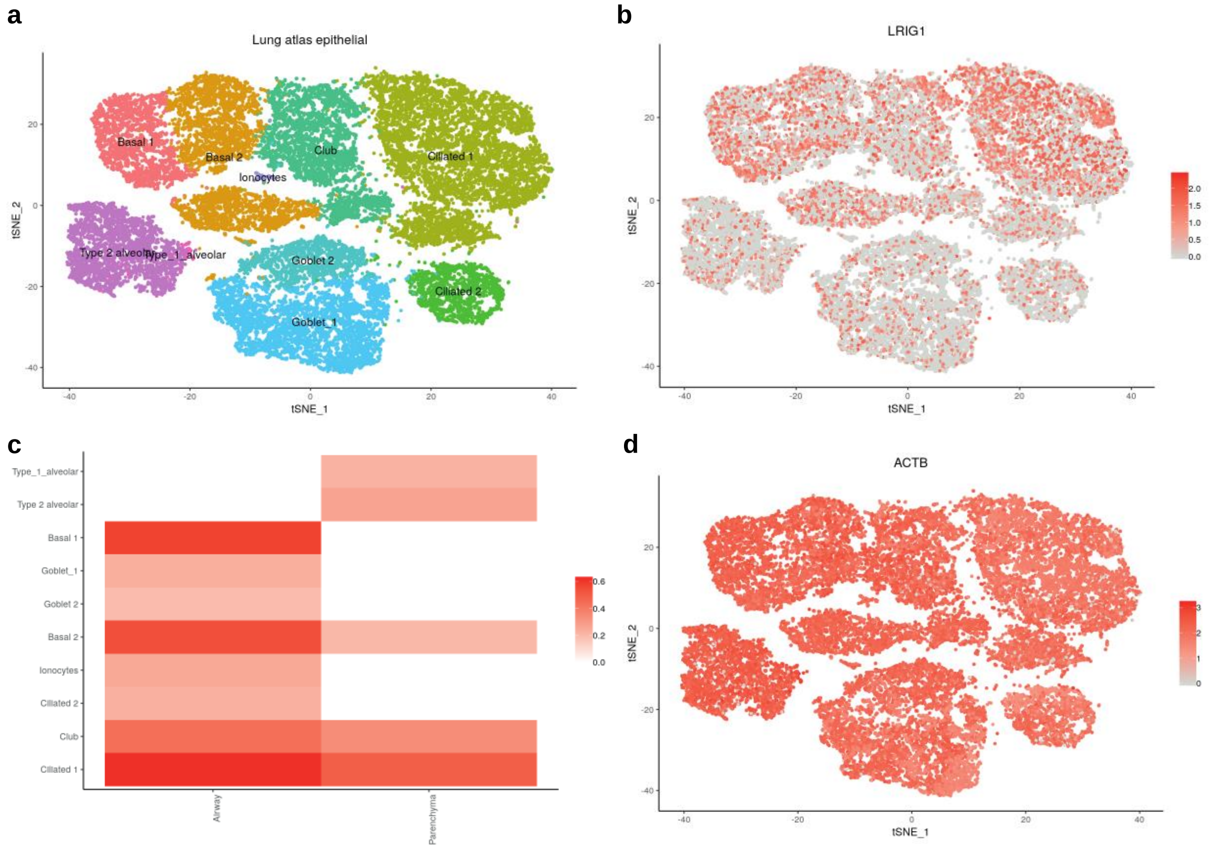

Supplementary Figure S2. Expression of *LRIG1* in the human adult airway epithelium. **(a)** Epithelial cell clusters present in the human airways. **(b & c)** Expression of *LRIG1* within the different human airway epithelial cell populations as assessed by single-cell RNA sequencing of 36 931 single cells from the human airways and lung parenchyma [10]. **(d)** Expression levels of the housekeeping gene *ACTB* are shown for comparison. Plots were downloaded from the www.lungcellatlas.org web portal.

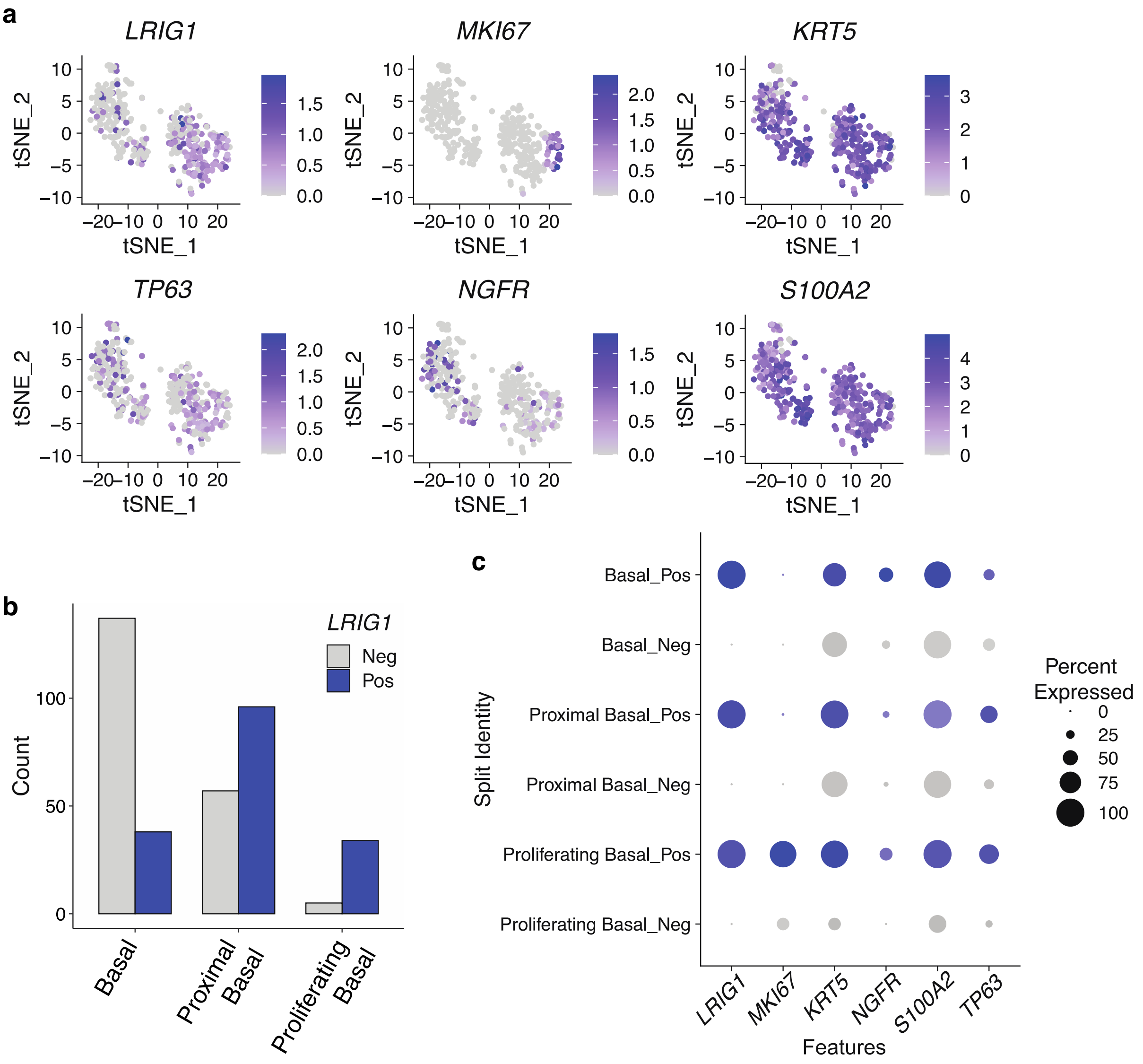

Supplementary Figure S3. Expression of *LRIG1* in bronchial basal cell clusters from the scRNAseq Human Lung Cell Atlas dataset. Normal human basal cells from bronchial epithelial tissue (*n*=367) were used to explore differences between the *LRIG1*$^{+}$ and *LRIG1*$^{-}$ subpopulations. **(a)** Feature plot displaying gene expression of *LRIG1* and selected marker genes in bronchial basal cells. **(b)** Count of cells with or without *LRIG1* expression in each basal cell cluster. **(c)** Dot plot depicting gene expression levels and percentage of cells expressing the indicated genes in *LRIG1* positive (Pos) or negative (Neg) basal cells.

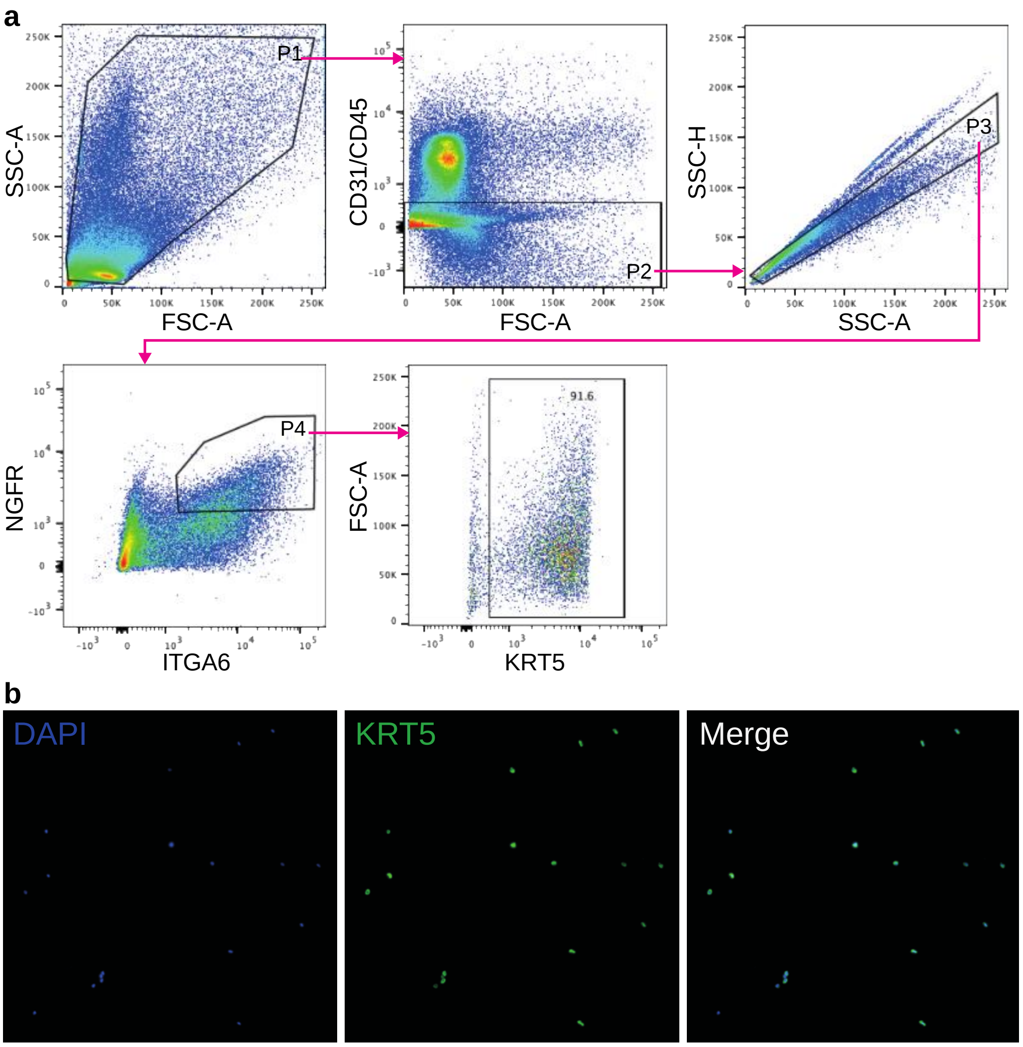

Supplementary Figure S4. Characterisation of flow-cytometry purified ITGA6^{+}NGFR^{+} human bronchial epithelial cells. **(a)** Following exclusion of debris, a cell population was selected (P1), from which both haematopoietic (CD45^{+}) and endothelial (CD31^{+}) cells were removed (P2). Single cells (P3) co-expressing ITGA6 and NGFR were then selected (P4). Within this double positive population, the fraction of cells expressing the basal cell marker KRT5 was assessed using intracellular staining. 85.6%±2.9 (mean±SEM, *n*=3 donors) of ITGA6^{+}NGFR^{+} human bronchial epithelial cells were KRT5^{+}. **(b)** Immunofluorescence staining for KRT5 in flow-cytometry purified ITGA6^{+}NGFR^{+} human bronchial epithelial cells.

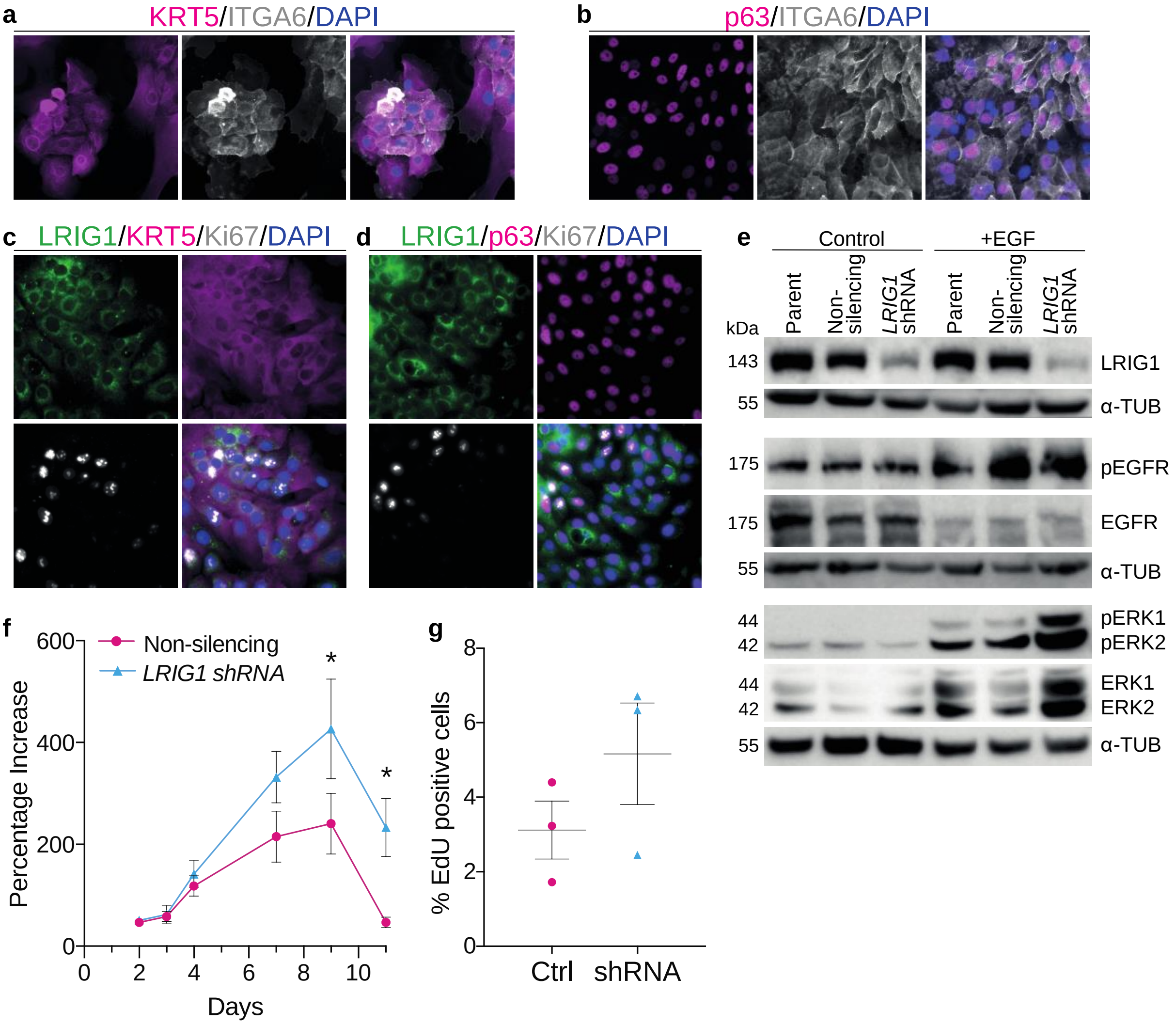

Supplementary Figure S5. LRIG1 is expressed in primary human bronchial epithelial cells (HBECs) *in vitro*. **(a, b)** Primary HBECs express the basal cell markers KRT5, ITGA6 and p63 *in vitro*. **(c,d)** Immunofluorescence staining for LRIG1, the basal cell markers KRT5 and p63, and the proliferation marker Ki67 in cultured HBECs. HBECs were transduced with lentivirus expressing either an shRNA against *LRIG1* or a control non-silencing construct. **(e)** Modified HBECs were starved for 16h and stimulated with or without 10 ng/mL EGF for 30 min. Immunoblot confirming successful *LRIG1* knockdown in cells carrying the *LRIG1* shRNA construct. *LRIG1* knockdown results in enhanced EGFR signalling activation. **(f)** XTT viability assays show enhanced expansion rate in *LRIG1* knockdown HBEC cultures when compared to controls (mean±SEM, n=4 donors). Two-way ANOVA: treatment effect (p=0.0016) and time effect (p<0.0001). *Sidak's multiple comparison test p<0.03. **(g)** EdU incorporation assay following *LRIG1* knockdown in HBECs (mean±SEM, n=3 donors). Ratio paired t-test (p=0.0599).

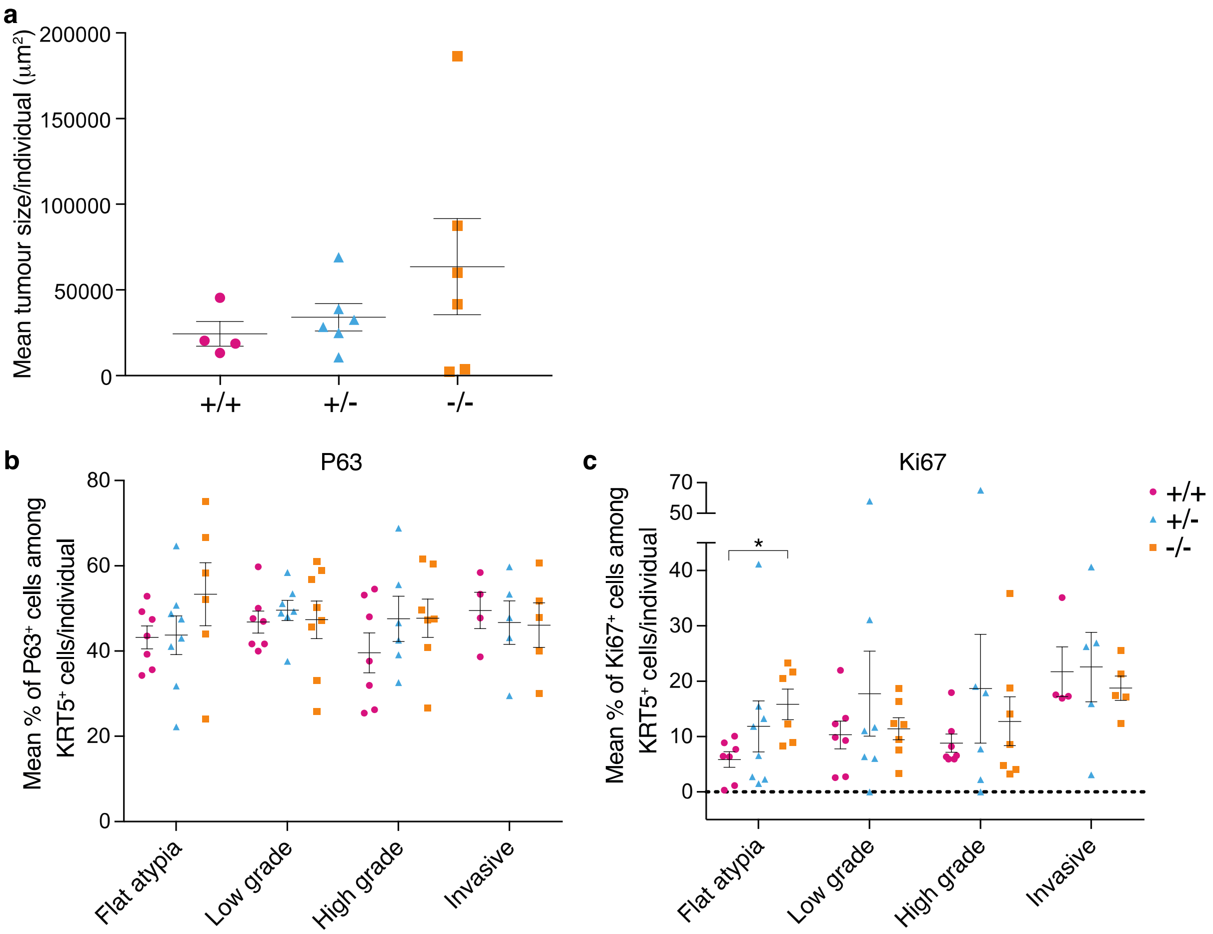

Supplementary Figure S6. Analysis of NTCU-induced pre-invasive lung lesions and tumours in individual mice of different *Lrig1* genotypes. **(a)** Average size of invasive LUSC tumours in individual wild-type (+/+), *Lrig1*-heterozygous (+/-) and *Lrig1*-null (-/-) mice treated with NTCU. Mean±SEM for individuals of the same genotype is also shown. The proportion of KRT5$^+$ cells expressing **(b)** P63 and **(c)** Ki67 in lesions of distinct grades was quantified. Dot plots represent average ratios in individual mice. For each lesion type, mean±SEM of values from mice of the same genotype is also shown. *Kruskal-Wallis test *p*=0.037, followed by Dunn's multiple comparisons test *p*=0.036.

SUPPLEMENTARY REFERENCES

1. Wang Y, Zhang Z, Yan Y, Lemon WJ, LaRegina M, Morrison C, Lubet R, You M. A chemically induced model for squamous cell carcinoma of the lung in mice: histopathology and strain susceptibility. *Cancer Res* 2004: 64(5): 1647-1654.
2. Stathopoulos GT, Sherrill TP, Cheng DS, Scoggins RM, Han W, Polosukhin VV, Connelly L, Yull FE, Fingleton B, Blackwell TS. Epithelial NF-kappaB activation promotes urethane-induced lung carcinogenesis. *Proc Natl Acad Sci U S A* 2007: 104(47): 18514-18519.
3. Tago Y, Yamano S, Wei M, Kakehashi A, Kitano M, Fujioka M, Ishii N, Wanibuchi H. Novel medium-term carcinogenesis model for lung squamous cell carcinoma induced by N-nitroso-tris-chloroethylurea in mice. *Cancer Sci* 2013: 104(12): 1560-1566.
4. Suzuki Y, Miura H, Tanemura A, Kobayashi K, Kondoh G, Sano S, Ozawa K, Inui S, Nakata A, Takagi T, Tohyama M, Yoshikawa K, Itami S. Targeted disruption of LIG-1 gene results in psoriasiform epidermal hyperplasia. *FEBS letters* 2002: 521(1-3): 67-71.
5. Wong VW, Stange DE, Page ME, Buczacki S, Wabik A, Itami S, van de Wetering M, Poulsom R, Wright NA, Trotter MW, Watt FM, Winton DJ, Clevers H, Jensen KB. Lrig1 controls intestinal stem-cell homeostasis by negative regulation of ErbB signalling. *Nat Cell Biol* 2012: 14(4): 401-408.
6. Hegab AE, Ha VL, Attiga YS, Nickerson DW, Gomperts BN. Isolation of basal cells and submucosal gland duct cells from mouse trachea. *J Vis Exp* 2012(67): e3731.
7. Butler CR, Hynds RE, Gowers KH, Lee Ddo H, Brown JM, Crowley C, Teixeira VH, Smith CM, Urbani L, Hamilton NJ, Thakrar RM, Booth HL, Birchall MA, De Coppi P, Giangreco A, O'Callaghan C, Janes SM. Rapid Expansion of Human Epithelial Stem Cells Suitable for Airway Tissue Engineering. *Am J Respir Crit Care Med* 2016: 194(2): 156-168.
8. Travaglini KJ, Nabhan AN, Penland L, Sinha R, Gillich A, Sit RV, Chang S, Conley SD, Mori Y, Seita J, Berry GJ, Shrager JB, Metzger RJ, Kuo CS, Neff N, Weissman IL, Quake SR, Krasnow MA. A molecular cell atlas of the human lung from single-cell RNA sequencing. *Nature* 2020: 587(7835): 619-625.
9. Butler A, Hoffman P, Smibert P, Papalexi E, Satija R. Integrating single-cell transcriptomic data across different conditions, technologies, and species. *Nature biotechnology* 2018: 36(5): 411-420.
10. Vieira Braga FA, Kar G, Berg M, Carpaij OA, Polanski K, Simon LM, Brouwer S, Gomes T, Hesse L, Jiang J, Fasouli ES, Efremova M, Vento-Tormo R, Talavera-Lopez C, Jonker MR, Affleck K, Palit S, Strzelecka PM, Firth HV, Mahbubani KT, Cvejic A, Meyer KB, Saeb-Parsy K, Luinge M, Brandsma CA, Timens W, Angelidis I, Strunz M, Koppelman GH, van Oosterhout AJ, Schiller HB, Theis FJ, van den Berge M, Nawijn MC, Teichmann SA. A cellular census of human lungs identifies novel cell states in health and in asthma. *Nat Med* 2019: 25(7): 1153-1163.
