# Supplementary material for: Lrig1 expression identifies airway basal cells with high proliferative capacity and restricts lung squamous cell carcinoma growth
Source: Eur Respir J. 2022 Mar 31;59(3):2000816. doi: 10.1183/13993003.00816-2020 (PMC8968013; doi:10.1183/13993003.00816-2020)
Supplement: Supplementary file 2 [file ERJ-00816-2020.Shareable.pdf]

EUROPEAN RESPIRATORY JOURNAL
ORIGINAL RESEARCH ARTICLE
L. SUCCONY ET AL.

SHAREABLE PDF

Lrig1 expression identifies airway basal cells with high proliferative capacity and restricts lung squamous cell carcinoma growth

Laura Succony[1,5], Sandra Gómez-López[1,5], Adam Pennycuick[1], Ahmed S.N. Alhendi[1], Derek Davies[2], Sarah E. Clarke[1], Kate H.C. Gowers[1], Nicholas A. Wright[3], Kim B. Jensen[4] and Sam M. Janes[1]

[1]Lungs for Living Research Centre, UCL Respiratory, University College London, London, UK. [2]Flow Cytometry Facility, Francis Crick Institute, London, UK. [3]Centre for Tumour Biology, Barts Cancer Institute, Queen Mary University of London, London, UK. [4]Biotech Research and Innovation Centre, University of Copenhagen; Novo Nordisk Foundation Center for Stem Cell Biology, DanStem, University of Copenhagen, Copenhagen, Denmark. [5]These authors contributed equally to this work.

Corresponding author: Sam Janes (s.janes@ucl.ac.uk)

Check for updates

Shareable abstract (@ERSpublications)
LRIG1 is lost in development of squamous cell lung cancers. This study shows that LRIG1 marks basal airway progenitor cells with high proliferative potential and regulates progression of pre-invasive squamous cell lung cancer. https://bit.ly/3AbPtY3

Cite this article as: Succony L, Gómez-López S, Pennycuick A, *et al*. *Lrig1* expression identifies airway basal cells with high proliferative capacity and restricts lung squamous cell carcinoma growth. *Eur Respir J* 2022; 59: 2000816 [DOI: 10.1183/13993003.00816-2020].

This single-page version can be shared freely online.

Copyright ©The authors 2022.

This version is distributed under the terms of the Creative Commons Attribution Licence 4.0.

Received: 23 March 2020
Accepted: 1 Aug 2021

Abstract

Background Lung squamous cell carcinoma (LUSC) accounts for a significant proportion of cancer deaths worldwide, and is preceded by the appearance of progressively disorganised pre-invasive lesions in the airway epithelium. Yet the biological mechanisms underlying progression of pre-invasive lesions into invasive LUSC are not fully understood. *LRIG1* (leucine-rich repeats and immunoglobulin-like domains 1) is downregulated in pre-invasive airway lesions and invasive LUSC tumours and this correlates with decreased lung cancer patient survival.

Methods and results Using an *Lrig1* knock-in reporter mouse and human airway epithelial cells collected at bronchoscopy, we show that during homeostasis LRIG1 is heterogeneously expressed in the airway epithelium. In basal airway epithelial cells, the suspected cell of origin of LUSC, LRIG1 identifies a subpopulation of progenitor cells with higher *in vitro* proliferative and self-renewal potential in both the mouse and human. Using the N-nitroso-tris-chloroethylurea (NTCU)-induced murine model of LUSC, we find that *Lrig1* loss-of-function leads to abnormally high cell proliferation during the earliest stages of pre-invasive disease and to the formation of significantly larger invasive tumours, suggesting accelerated disease progression.

Conclusion Together, our findings identify LRIG1 as a marker of basal airway progenitor cells with high proliferative potential and as a regulator of pre-invasive lung cancer progression. This work highlights the clinical relevance of LRIG1 and the potential of the NTCU-induced LUSC model for functional assessment of candidate tumour suppressors and oncogenes.
